# Comprehensive perceptions at the interface between health and environment: Applications models with a citizen science tool

**DOI:** 10.1192/j.eurpsy.2025.10107

**Published:** 2025-11-20

**Authors:** Frauke Nees, Karina Janson, Stephan Lehmler, Sarah Böttger, Mira Tschorn, Philipp Hummer, Gunter Schumann, Michael Rapp, Sebastian Siehl, Nathalie Holz, for the environMENTAL Consortium

**Affiliations:** 1 Institute of Medical Psychology and Medical Sociology, University Medical Center Schleswig-Holstein, Kiel University, Kiel, Germany; 2 Department of Child and Adolescent Psychiatry and Psychotherapy, Central Institute of Mental Health, Medical Faculty Mannheim and University of Heidelberg, Mannheim, Germany; 3 Social and Preventive Medicine, Department of Sports and Health Sciences, University of Potsdam, Potsdam, Germany; 4 SPOTTERON Citizen Science, Vienna, Austria; 5 PONS Research Group, Department of Psychiatry and Psychotherapy, Campus Charite Mitte, Charité Universitätsmedizin, Berlin, Germany; 6 Institute for Science and Technology of Brain-inspired Intelligence (ISTBI), Fudan University, Shanghai, PR China; 7 German Center for Mental Health (DZPG), partner site Mannheim-Heidelberg-Ulm, Mannheim, Germany

**Keywords:** citizen science, environment, mental health, precision medicine, psychogeography

## Abstract

**Background:**

Lived experience – how individuals perceive and interact with their environment – plays a central role in understanding mental health. Yet, insights into this first-person perspective, including subjective thoughts, emotions, and socio-contextual influences, remain limited in current research approaches.

**Methods:**

To address this gap, we developed *StreetMind*, a scalable, secure, and user-friendly digital citizen science platform grounded in a psycho-sociogeographic framework. The platform collects self-reported data on individuals’ activity spaces through a mobile app and web interface, capturing location visits, travel routes, and daily experiences. These subjective reports are combined with objective real-time health, environmental, and sociocultural data to generate integrated community “footprints.”

**Results:**

Initial usage data (*N* = 1,010 for location and route entries; *N* = 509 for daily experiential data) demonstrate the platform’s structural robustness and functional feasibility. *StreetMind* enables classification of daily experiences by linking personal perceptions with contextual environmental data. This integration facilitates the identification and quantification of key environmental and psychosocial factors associated with mental well-being.

**Conclusion:**

*StreetMind* offers a novel, data-rich mapping of health–environment interactions by merging individual lived experience with environmental metrics. This approach supports the creation of dynamic “health–environment spaces” and holds promise for informing public health strategies and advancing precision mental health care.

## Introduction

Mental and psychophysiological disorders are among the most significant causes of years lived with disability [1]. Even with optimal care, <30% of the burden from mental disorders can be alleviated, highlighting the need to better understand factors that influence health and well-being. Despite decades of research, many gaps remain in our understanding of how health is shaped by the environment, which hampers advancements in precision medicine – an approach aiming to develop personalized strategies for individuals and the public health system. Among recognized determinants of health, the integration of information regarding environmental factors is becoming increasingly important. Environmental factors are increasingly recognized as central determinants of health. Recent tools in GPS tracking and citizen science have explored dynamic environmental exposure, yet few have combined subjective and objective real-time data with individual interaction and perception at this level [2–8]. Our physical surroundings – where we live, learn, work, play, and socialize – shape daily behaviors, experiences, and emotions. The World Health Organization and numerous studies [9, 10] estimate up to 20% of mental health conditions, such as depression and anxiety, can be attributed to physical environmental influences.

A growing body of research has connected mental health and environmental factors, including urbanization, climate change, and access to nature, with proximity to green spaces exerting positive effects on health [9–16]. Access to and contact with nature, with green and blue spaces such as forests, lakes, and coastlines, have been associated with improved mental well-being [2, 3], possibly due to psychological connectedness to nature [17]. However, many of these studies rely on satellite data [3, 4] and lack insights into how individuals actively engage with their environments, limiting conclusions about underlying mechanisms [5].

To address this, new tools are needed that go beyond passive tracking (e.g., GPS logging [18, 19]) to capture individuals’ interactions with their environments, including detailed travel diaries, user ratings, or contextual experiences. These methods can help visualize lived experiences and provide more meaningful data on environmental exposure through participatory approaches such as citizen science [20–22]. Rather than focusing solely on proximity, we must also consider the quality and nature of how people engage with environments. In general, environmental factors can impact health both directly – for example, through prolonged noise or heat exposure causing physical harm – and indirectly through stress, particularly when environmental demands exceed an individual’s ability to cope [23]. While many studies have explored the influence of stress and annoyance in this context [11, 24, 25], there is still limited understanding of the cumulative impact of complex environmental exposures on both physical and emotional outcomes.

Importantly, the effects of environments are shaped not only by objective characteristics but also by subjective perception [26]. Theories like the stress appraisal model by Lazarus and Folkman [27], the attention restoration [28, 29], the stress reduction theory [30], and the biophilia hypothesis [31] emphasize that stress arises when there is a mismatch between situational demands and an individual’s perceived capacity to cope, and that natural environments can help alleviate stress. Even in the absence of physical harm, environments can negatively impact mental health if they are perceived as demanding or unsafe. Understanding how movement patterns intersect with environmental experience may offer valuable insights for personalized prevention strategies. Although prior research has considered individual competencies, physiological characteristics, lifestyle, urban land use, development density, transportation systems, and other aspects of the built environment [32–34], there remains a lack of integration between subjective rating, passive data, and environmental context. This disconnect can lead to overly general interventions that miss individual needs.

Emerging tools like UrbanFootprint [6] and OpenStreetMap (OSM) [7] offer precise spatial data, which, when combined with subjective user input, provide a deeper view of individual health–environment interactions. Mapping routes and frequently visited places (“spots”) with individual ratings reveals what people notice, value, or avoid, making it possible to identify emotional triggers or restorative locations. This can support more effective and personalized prevention and intervention strategies.

To advance this, we must move beyond lab-based models and gather data that are more closely related to everyday life, which is possible with digital tools [8]. Smartphone apps enable ecological momentary assessments (EMAs) to capture feelings, emotions, and health-related aspects throughout the day [35–38], and can be used as digital travel diaries as well [39]. Yet, we also need to expand beyond traditional tools in epidemiology and environmental research that focus on logistics, freight efficiency, or broad infrastructure mapping (e.g., BetterFootprint/SmartWay [40], N-PHAM [41], and EnviroAtlas [42]). By involving individuals more actively via citizen science [43], we can make health and environmental monitoring more transparent, interactive, and scalable. This participatory approach helps ensure interventions remain relevant and meaningful for users, while also serving as a resource for policy and public health planning [44–46]. This ensures that interventions are both meaningful to individuals and scalable to populations.

To address these challenges, we developed *StreetMind* (https://www.streetmind.eu), a modular digital citizen science platform at the nexus of environmental exposure and mental health. *StreetMind* allows for dynamic mapping of (daily) behaviors and emotional states in relation to physical surroundings. In addition to passive data collection like location tracking, it integrates self-relevant information and activities about regular paths, places, and perceptions. By combining these insights with environmental and demographic information, *StreetMind* supports a psycho-sociogeographic citizen science approach to precision health. It bridges disciplines – from clinical and cognitive science to urban planning – to develop cost-effective, scalable assessments for real-world health monitoring and intervention design.

Our research integrates both subjective and objective measures and assesses multiple environmental co-factors. We hypothesize that perceptions and exposures interact at different levels to influence mental health. In this manuscript, we present application models of the platform using data from a pilot sample, demonstrating how individual movement, perception, and context shape daily behaviors and mental well-being.

## Methods

### *StreetMind:* Modular functionality, assessment concepts, and citizen science principles

#### Overview and modular design

*StreetMind* is a modular and user-adaptable assessment platform, comprising a smartphone app (Android: https://play.google.com/store/apps/details?id=com.spotteron.streetmind; iOS: https://apps.apple.com/us/app/streetmind/id1642341183) and a website (https://www.streetmind.eu), designed to capture subjective and objective information about individuals’ lived environments, experiences, and well-being in a scalable and personalized way, which reflects the dynamics of societal and healthcare challenges [47]. Its structure supports active and passive data collection, combined with citizen science engagement tools and both app-based and web-based interfaces. It can be applied in a wide range of applications, from small-scale empirical and clinical studies to large epidemiological cohorts and community engagement.

**Figure 1.**
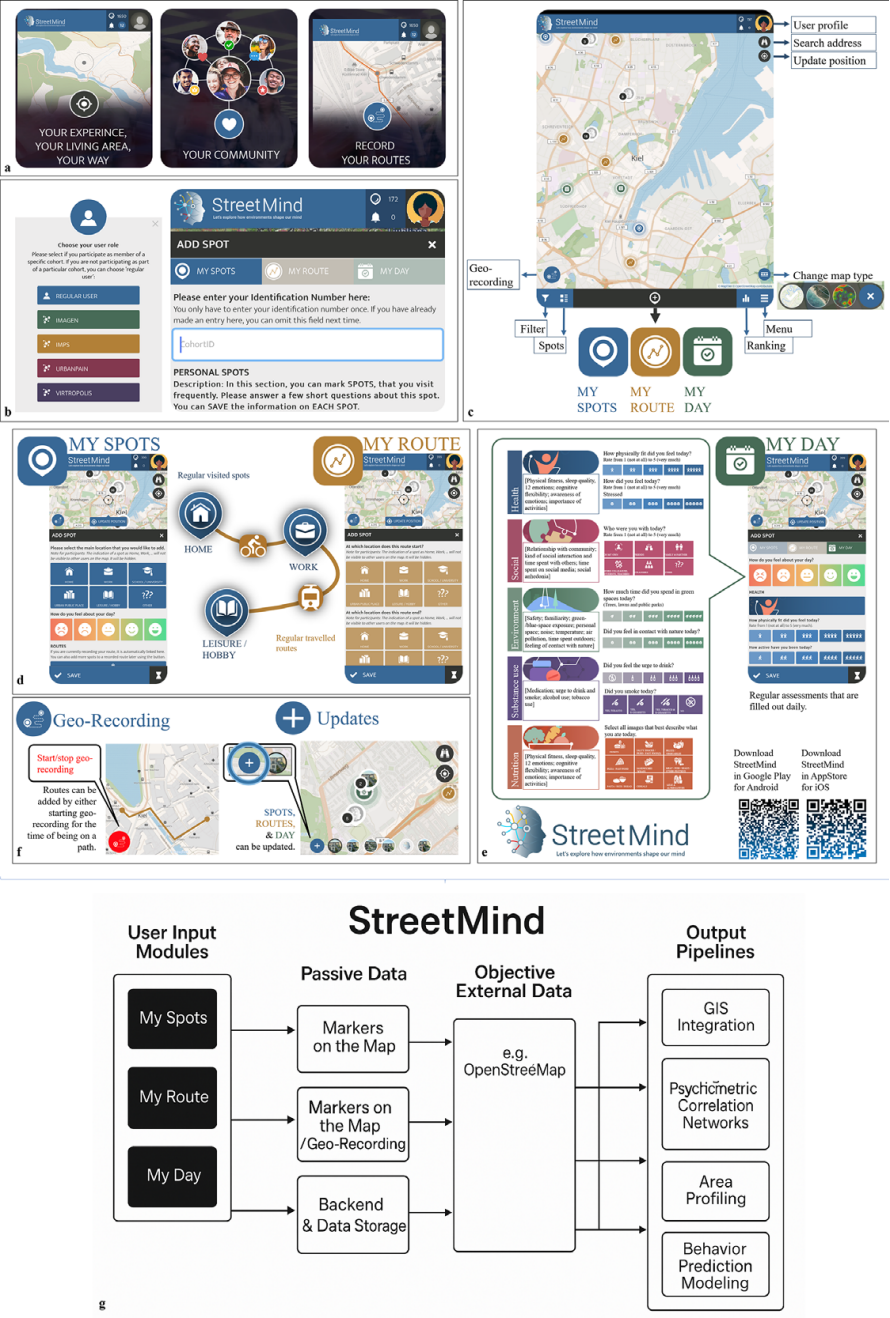
*StreetMind* app visualization, functions, and flowchart. Registration components and core features: (a) Introduction slides; (b) user role and user code; and (c) general app components. Modules: (d) Information on spots and routes (where and how are individuals moving through and behaving in their environment and how they think and feel about it); (e) daily specificities on health-related states and behaviors; (f) geo-recording, updating, and adding of special circumstances and events; and (g) flowchart illustrating the modular structure and data flow in *StreetMind.*

The *StreetMind* app (Figure 1a–f) allows users along flexible pipelines (Figure 1b) to post frequently visited spots, traveled upon routes, and daily emotions and behaviors in their environment (Figure 1a,c). The *StreetMind* website provides a survey tool for participants to complete validated questionnaires. *StreetMind* is user-friendly, interactive, and fosters citizen science and capturing dynamic real-world experiences, aligning with calls for technology in health care to prioritize public benefit over private profit [48]. Additionally, it aims to explore citizens’ perceptions of the challenges and ethical considerations inherent in research and innovation (“innovation dilemmas”), such as ensuring data privacy and balancing societal benefits with commercial objectives [49]. The platform comprises three main functional domains: spatial, temporal, and socio-emotional assessment, presented through modular subcomponents (Figure 1d–f), and accessible either via app or browser using a unified user account system.

#### App features and module overview

- Assessment modules: Spatial, temporal, and socio-emotional contexts

*StreetMind* contains three core user-facing modules – *MySpots*, *MyRoute*, and *MyDay* – which collect both structured self-report and passively recorded data across different domains:

- “*My Spots*” allows users to indicate frequently visited locations using geo-positioning and annotate each location with structured questions on the purpose of the location (e.g., work), environmental factors (e.g., closeness to green/blue spaces), social aspects (e.g., social connectedness), physical activity and well-being, evoked emotions, and mental well-being (Figure 1d; Supplementary Table 1).
- *My Route* enables users to draw or track routes they frequently travel and respond to similar questions as in *My Spots.* Both modules support active marking and passive GPS recording, and entries can be updated over time. These spatial records (spots and routes) can be made either with or without real-time tracking (Figure 1f).
- *My Day* captures daily subjective experiences and behaviors using an EM structure. Questions are grouped into five different sections: physical and mental health, social aspects, environmental factors, substance use, and nutrition (Figure 1e; Supplementary Table 1). Items are adapted from the Research Domain Criteria domains, including social processes and positive and negative valence [50].

**Figure 2.**
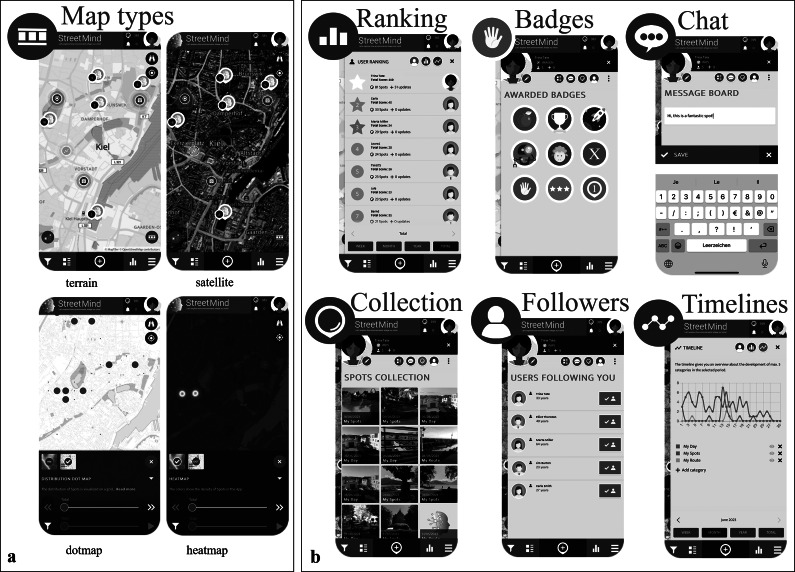
*StreetMind* citizen science related features: (a) Advanced graphical solutions (here: different map types for spots, routes, and ratings as examples) and (b) interactive and motivational tools (here: examples of social [rankings, chats, and followers], performance-related [rankings], environment-related [pictures], and rating-related [timelines of entries] aspects).

These modules are equipped with citizen science features (see also below; Figure 2), including optional feedback, interactivity, and engagement tools, to foster engagement and interaction throughout the assessment process.

1. Combination of objective and subjective information and daily specificities

*StreetMind* enables the construction of individualized network models of participants’ lived environments through the integration of:

- Subjective entries (e.g., emotional valence and perceived social connectedness),
- Objective geo-data (e.g., GPS, activity tracking, and physical distance between nodes),
- Temporal context (e.g., daily variation via *MyDay* EMA).

Each user’s environment is thus modeled as a spatial network of nodes (spots) and edges (routes), allowing for both within-person and between-group analysis of how emotional experience, place characteristics, and behavioral patterns interact.

These multilayered data enable investigation of both stable and context-dependent influences on health and well-being. For example, emotional ratings and social connectedness in My Spots, My Route, and My Day can be correlated with movement patterns and neighborhood features captured through passive recordings.

1. Platform flexibility and adaptability

The modules serve as the building blocks of *StreetMind*, supporting modular reconfiguration and allowing different combinations of feature sets across research studies and user groups. All modules are embedded into a platform-wide environment where projects can be managed independently while sharing common infrastructure and data formats (Figure 1b). Contents can be changed depending on the study, sample, or consortium, without changing the platform. This allows quick implementation and adaptation for new studies or cohorts, including cross-national or longitudinal studies, while keeping inter-study compatibility and reuse of existing pipelines.

1. Citizen science principles and interactive features

*StreetMind* is based on citizen science principles, that is, research for the people, with the people, and by the people [46], and so promotes co-creation, transparency, and public engagement:

- Interactive elements include map-based visualization (e.g., switchable layers in Figure 2a), chat functions, blog updates, and customizable feedback (details can also be found in Supplementary Material 1.2).
- Individual feedback is provided through heat maps visualizing aggregate or personalized emotional/environmental data over time. These highlight positive or problematic areas (e.g., low well-being zones and perceived noise exposure) and how these shift longitudinally.
- Gamification tools, including badges (e.g., for first entry), user rankings, and timelines, enhance engagement and reinforce sustained participation (Figure 2b).

These features aim to empower users, foster scientific understanding, and contribute to community-level knowledge transfer across disciplines.

The *StreetMind* platform was designed and developed together with the SPOTTERON Citizen Science App Platform (https:///www.spotteron.net), who provide visual design, technical development, IT infrastructure, and support to run both applications.

1. StreetMind web interface and psychometric and trait-level assessment

The *StreetMind* website (https://www.streetmind.eu/) complements the app and includes access to standardized questionnaires, used in studies for basic trait-like sample characterizations. An example of a currently used survey includes questions about socio-demography, urbanicity, eco-anxiety, personality, physical and mental health, and health-related behaviors (reward, inhibition, and intention; Supplementary Table 2). Projects can select and configure these instruments per study using user-role tagging. Web-inclusive features include newsletter subscription, blog function, and optional data contributions via the browser.

1. Privacy, data protection, and data management

Participants can choose to save information on spots and routes to be visible to other users, who can interact with them by, for example, liking or commenting on a spot. They can also choose which entries will be visible to other users and decide when they want to accept geolocation-recording (not set in a 24 h mode). Very sensitive information (e.g., spot links to home/workplace and sensitive information in the *My Day* section will never be made visible to others. SPOTTERON also provides data management tools (https://www.spotteron.net/citizen-science-app-features/data-handling) and data are stored on secure servers at the Institute of Medical Psychology and Medical Sociology, University Medical Center Schleswig-Holstein in Kiel. More information on privacy settings, data protection, and data management can be found in the Supplementary Material 1.1.

1. Pilot use case and application feasibility

We present the first data and analyses to demonstrate the feasibility and application potential of the *StreetMind* app, according to factors on objective and subjective indicators of the environment and its link to individual health. The application models are based on *N* = 1,010 entries to the *My Spots* and *My Route* and *N* = 509 for *My Day* from a community sample in Kiel (58.8% female, age = 32.5 years [SD = 13.65]), captured over a period of 4 weeks with *My Day* questions being asked one time per day (evening: “How was your day?”). Eligible individuals were 20–40 years old, resided within Kiel city limits, owned an Android (v9+) or iOS (v14+) smartphone with a mobile data plan, and consented either (a) to share background GPS data, which they could start or stop at any time, or (b) to enter their locations manually on the in-app map. Inclusion criteria were age between 18 and 65 years and sufficient knowledge of the German language. Exclusion criteria included any current or pre-existing neurological or psychiatric condition, intake of psychopharmacological medication, no smartphone, and no regular internet access.

For data analyses, we used R (version 4.3.1, https://www.r-project.org) and Python (version 3.12.0, https://www.python.org) (see Supplementary Table 3 for detailed technical methods, code, and spatial analysis pipelines). Individual ratings of regular spots, routes, and daily assessments, alongside day-specific details, served as subjective information. These elements are crucial individual co-determinants indicating how personal factors shape experiences. To ground subjective data in an objective context, we enriched this data with information gathered from OSM, which also allows us to link individual information from the app to information on the built environment elements collected by the tool. Geospatial objects in *StreetMind* are stored as either GeoJSON objects or Latitude/Longitude pairs in World Geodetic System 1984 format to ensure precise location tracking. For a deeper understanding of accessibility and connectivity in participants’ environments, we extracted the street networks from OSM and calculated network centrality measures for street segments and whole streets using street names in OSM, which allows the analysis of how the connectedness of streets might influence participant experiences and perceptions. We further calculated the spatial distance between points marked on the map (individual spot entries) using the spherical haversine approximation. This distance analysis was used to assess the spatial distribution of activity spaces, allowing for insights into how the geographic spread of participants’ environments might impact their well-being. To explore relationships between subjective health and environmental ratings, we calculated correlation networks using polychoric correlations for ordinal variables (derived from a 5-point Likert scale). We then used spider charts to compare daily ratings by location by calculating the frequency of attributes used to describe places within and outside a predefined bounding box (covering the city of Kiel) and normalizing for the number of points observed. Finally, we estimated spatial and variable-specific densities to identify areas and experiences that are most frequently rated or reported. For spatial density, we applied an axis-aligned bivariate normal two-dimensional kernel for the estimation of, and for the other variables, we used a one-dimensional Gaussian kernel in the ridgeplots. These density estimates highlight “hotspots” of activity and emotional responses, allowing us to pinpoint where certain experiences are concentrated and how participants’ environments might shape their perceptions.

## Results

### Validation of response variability across the app items

**Figure 3.**
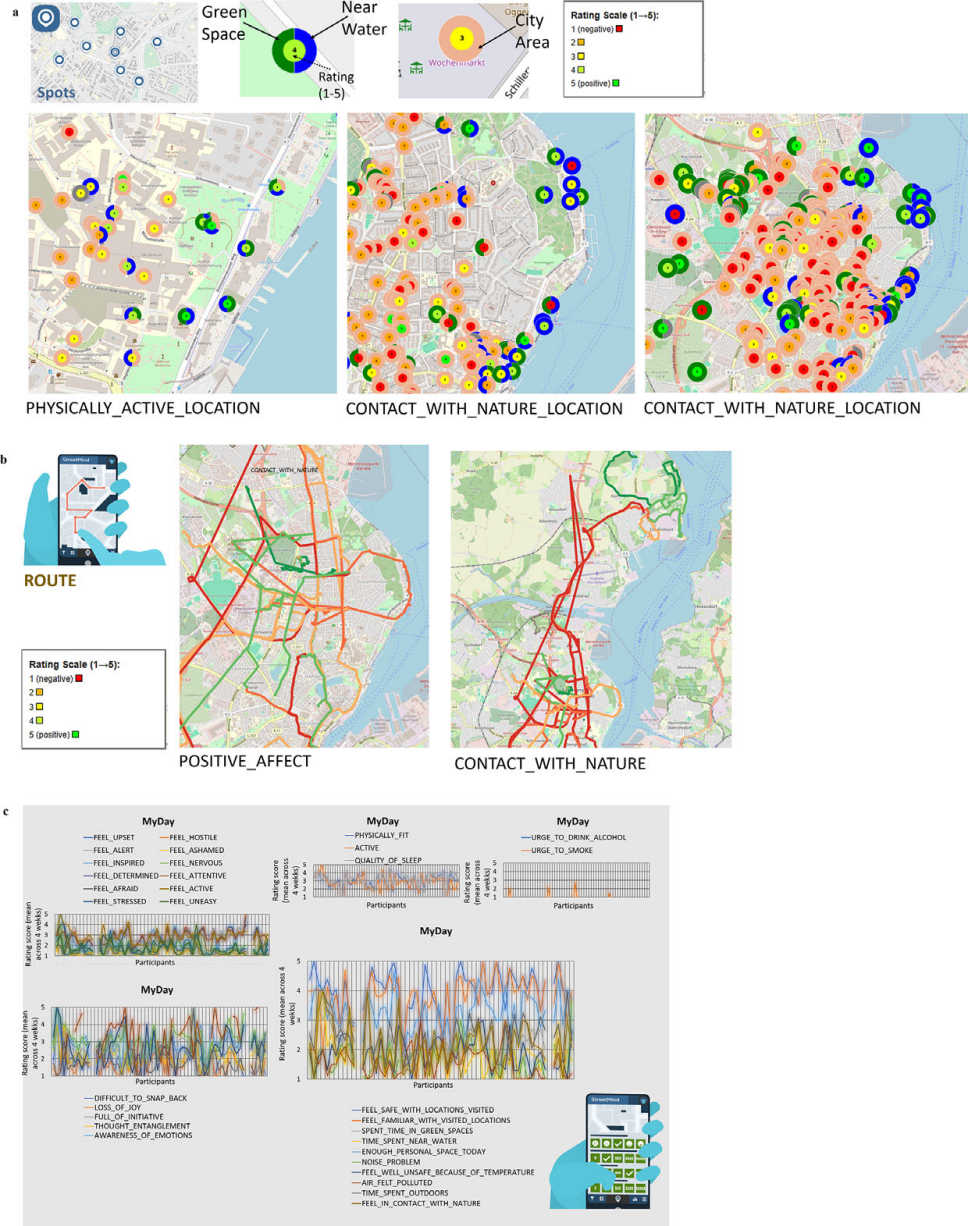
Overview of variation between individuals in the rating data across items *My Spots*, *My Routes*, and *MyDay* module from *StreetMind.*

We can demonstrate very good variability among the participants regarding the different items from the *StreetMind* modules, enabling targeted analyses for different projects (see Figure 3).

Into fine-grained subjective information and variations of individual affect and perceptions of and behaviors in the environment

**Figure 4.**
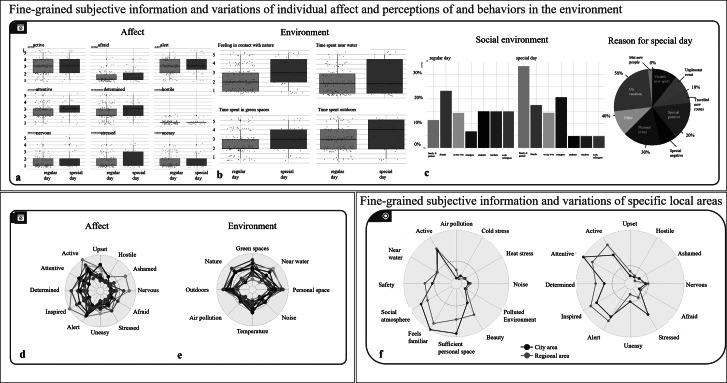
*StreetMind* exemplified application models for (a–f) fine-grained subjective information and variations of individual affect and perceptions of and behaviors in the environment (here: mood ratings depending on regular or specific days, information on environmental exposure during regular or specific days, mood- and environmental-related networks, and internal and external co-determinants with respect to specific characteristics of regular and special days); and (g) fine-grained subjective information and variations of specific local areas (here: environmental feature-related networks for city and regions areas).

To illustrate how individual ratings of health and environment are influenced by the specific characteristics of each day, we plotted the overall distribution and variations in mental states (Figure 4a,e [affect]) and environmental engagement, like feelings or time spent in specific settings (Figure 4b,f [environment]). We then linked these ratings to whether a day was regular or special, along with its specific characteristics, including the social environment (Figure 4c) and the reasons for special days Figure 4d), which is reflected in partially different affect- and location-related environmental ratings (Figure 4a,b). While affect is rather similar for regular and special days, this is not the case for environmental perceptions and behaviors, indicating that the subjective perceptions of the environment are important. It shows that an objectively favorable location, such as green space, can have markedly different subjective effects depending on various individual circumstances, or may even not be the driving force for health and related behaviors. From our pilot data, we, for example, see that particularly time spent in nature and in green spaces marks the subjective experience of whether a day is special. Even a single side factor, like whether it is a regular or special day, can thus make a significant difference. In addition to the physical environment, special days are also characterized by changes in the social environment of participants – for instance, an increase in interactions with the family members. This may be a critical co-determinant or even driving force of health–environmental interactions, and various reasons stand behind the classification of a regular or special day. Thus, *StreetMind* captures the contextual factors that shape well-being beyond momentary emotional states. This opens up a window into new and so far hidden factors, which are important both to investigate specific types of days to improve data modeling and to avoid potential bias with respect to factors triggering health. It also adds increased temporal granularity by considering leading to potential varying emotional, social, and environmental experiences throughout the day.

While having focused on the novel aspect of *StreetMind*, the description of daily experiences, including health items, can be analyzed on any other granularity suitable for specific research questions. For example, by not only capturing both objective patterns and subjective nuances, but also according to commonly used EMAs in health-related studies or map-based solutions used for environmental and epidemiological studies, including urban planning and distributions of health- and environmental-related risk factors for the population.

### Into fine-grained subjective information and variations of specific local areas

Integrating geo-referenced location markers into the models, we illustrate a targeted focus on specific areas and the creation of multidimensional area profiles in relation to both subjective and objective health indicators. Here, we can see that both ratings on environmental features and affect differ when comparing urban and rural settings (Figure 4g). While rural areas are described more in terms of positive visual features, such as beauty and nature, city areas are more strongly characterized in terms of interactive features. These profiles are reflected in indicated affect dimensions, with a rural area felt as being more inspiring and, thus, rather reflecting the so-called “hot” cognitive functions that are related to emotions and reward, while a city area fosters more so-called “cold” cognitive processes such as increased attention. Moreover, there is a divergent association between environmental and affect ratings for these two areas, indicating not only that each area may foster a different engagement, but also that specific subjective characteristics of an environment are differently linked to the self-relevant affect-related indicators of health depending on the objective characteristics of an area (here: rural and city area). The integration of static location-bound data with dynamic information enables a more granular capture of both subjective and objective mental processes and interactions within an individual’s environment. This approach facilitates a nuanced modeling of environmental differences and linking these to health-related behaviors or long-term effects of environmental interactions.

### Into “hotspots” of subjectively described environments and the link to their objective markers

**Figure 5.**
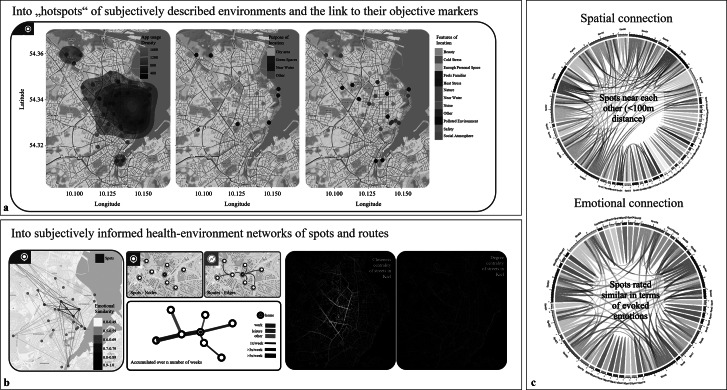
*StreetMind* exemplified application models for (a) “hotspots” of subjectively described environments and the link to their objective markers (here: heat maps of location-specific densities and area and spot characteristics); (b) subjectively informed health-environmental networks of spots and routes (here: examples of networks of behavioral pathways as an interaction between routes, spots, transportations, and individual ratings); and (c) spatial (here: based on distance measures) and emotional connections (here: based on ratings).

In addition to characterizing specific areas, we also zoomed into user-marked spots, examining detailed subjective and objective data, such as emotional responses, environmental descriptors, and functional attributes. By summarizing key variables and activity levels as heatmaps, we gained insights into how different spots are subjectively perceived and whether there is alignment with broader objective categorizations, such as whether a spot is in an urban area, near green spaces, or close to water (Figure 5a). The discrepancies in these objective categorizations underscore the usefulness of having additional self-relevant, subjective information. This allows us to assess whether these broader environmental categories are consistently reflected in individual perceptions or if there is a variability between objective characteristics and subjective impressions. This unique combination of spatial and psychological data can be expanded using further information, such as pictures from *StreetMind.* Linking physical features to mental states in future studies allows for a granular decomposition of physical space and its contribution to health.

### Into subjectively informed health–environmental networks of spots and routes

We further integrated spatially linked data with subjective daily experiences, routes, and locations. This creates a “health space” map that connects emotional networks (how people feel in specific places) with individual spatial-cognitive maps (how people perceive and organize these places mentally). Thereby, it can be inferred how specific spots are emotionally linked – that is, whether they elicit similar feelings – and how individuals represent these spots in terms of spatial distance or closeness to determine whether individuals differ in the number and diversity of such networks (Figure 5b). By continuous tracking over several weeks, these data enable us to assess whether these emotional-geographic networks are stable over time and how frequently they occur. This enables building transit maps (“footprints”) that show movement patterns across specific areas, such as a city, and associating these patterns with mental health risks or resilience. Therefore, we modeled the spatial proximity and emotional similarity of the individual spots, creating a multidimensional view of the environment network of the Kiel area (Figure 5c). This allows us to see whether physically close spots are also rated emotionally similar and to identify “hotspots” of well-being and mental health risks.

### Interactions between daily subjective emotions, internal feelings, and environmental perceptions

**Figure 6.**
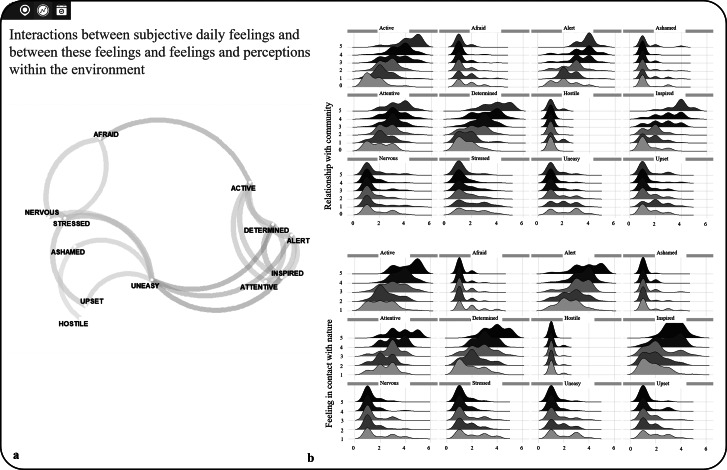
*StreetMind* exemplified application models for (a) interactions between subjective daily feelings and (b) interactions between these feelings and feelings and perceptions within the environment.

We explored the relationship between subjectively reported environmental, psychological, social, and physiological factors, both for daily affect and between affect and environmental perceptions. A correlation network was created, showing how different emotions co-occur together during the day, which can be valuable for understanding well-being and targeting specific emotional states in interventions (Figure 6a). As such, emotions are interconnected in distinct positive and negative clusters, with positive emotions tending to co-occur and negative emotions similarly grouped. The inverse relationship between positive and negative emotions suggests that certain mental states can help buffer against others (e.g., feeling determined and altered may reduce feelings of unease). This allows us to identify factors of well-being and see whether these patterns are shaped by different environments or occur independently. These analyses can be conducted on an aggregated level, as shown here, but could also be used for group comparisons or longitudinal individual-level assessments, offering insights into how emotional states evolve over time within unique contexts.

Additionally, we have modeled how different feelings are related to specific perceptions of connectedness to social and physical environments (Figure 6b). With increasing connectedness, the emotional ratings follow similar patterns across the physical and social environment, with higher levels of activeness, alertness, and inspiration. This is interesting because it shows that social and nature-based experiences have similar effects on individual well-being. This insight is highly relevant for health research and the development of prevention and intervention strategies, as it suggests that both the social and the physical environment should be considered together.

## Discussion

Our health is influenced by the world around us – spanning built, socioeconomic, chemical, physiological, and psychosocial dimensions. These interconnected domains collectively impact our well-being [48, 49, 51]. A central challenge in environmental health science is determining how to assess this multifaceted interplay – not only at the population level, but in ways that capture individual differences in both health and environmental experience. While traditional environmental health research focused on identifying and reducing environmental hazards [50], recent shifts have incorporated beneficial exposures such as access to green space, clean air, or supportive social environments, especially at the local level.

This study introduces *StreetMind*, a modular, multimodal citizen science platform designed to capture cohort- and community-level information while enabling fine-grained insight into the relationship between daily behavior, health, and environmental exposure. By integrating subjective experiences with objective data through a psycho-sociogeographical approach, *StreetMind* aligns with recent efforts in environmental psychology and geographic information science to better understand human–environment interactions [52, 53]. Compared to existing GPS-based or mobile sensing platforms, StreetMind offers several key advances. Traditional mobile-sensing studies have focused on passively capturing location, accelerometry, and app usage data to infer behaviors and mental states [54, 55]. While these methods provide continuous and objective data streams, they often lack contextual specificity and do not account for subjective perception. *StreetMind* fills this gap by combining passive sensing with self-initiated emotional, cognitive, and environmental annotations. It also incorporates participatory design and feedback mechanisms rooted in citizen science principles, making it not only a data collection tool but also a platform for user reflection, empowerment, and engagement.

Our findings underscore *StreetMind*’s value for capturing real-world complexity in mental health. For example, participants’ reported affective states often remained consistent between regular and special days, even when environmental and social contexts shifted significantly (e.g., more time in nature or with family). This suggests that context matters in nuanced, nonlinear ways and can differentially influence well-being across individuals and situations.

Moreover, the analysis of correlation networks between subjective experiences and environmental perceptions expands prior findings on mood-mapping and person–place interactions [56, 57], offering a network-based understanding of emotional co-regulation across space and time. These emotional networks, particularly when linked to specific geographic “hotspots” and activity routes, provide a multidimensional view of how individuals perceive and navigate their environments. This insight is critical for identifying “mental landscapes” that reflect personal preferences, triggers, and stressors, which could be highly relevant for developing personalized intervention strategies that target specific contexts and emotional patterns.

One of *StreetMind*’s unique contributions is, thus, the ability to contrast and integrate subjective perceptions of environments – such as perceived safety, beauty, or noise – with objective environmental features, such as green space proximity, urban density, or traffic volume. While objective environmental indicators provide standardized metrics and are crucial for policy and infrastructure design, subjective perceptions often better predict psychological outcomes like stress, mood, and social connectedness [53, 57]. Our findings underscore that these two domains – subjective and objective – may have independent, additive, or even interactive effects on health outcomes. For example, even when living near a park, individuals who perceive the area as unsafe or unpleasant may not experience its mental health benefits. Conversely, a person might find psychological relief in an objectively less green space due to personal attachment or positive memories. These insights support the argument for multilevel models that integrate both perspectives, enabling more nuanced prediction of mental and social health. This dual-perspective approach underscores the necessity of multilevel models that integrate physical and psychological features of environments. It also emphasizes the relevance of *StreetMind* in advancing precision mental health strategies by illuminating how context-specific experiences shape emotional well-being.

In addressing common challenges in longitudinal experience sampling, *StreetMind* captures both typical and atypical daily events. For instance, while some behavioral outliers may initially appear as noise, they may carry significant health-relevant insights, particularly when seen in relation to individual variability. The platform enables the capture of episodic events and daily fluctuations that might otherwise be missed by coarse sampling or retrospective reporting.

*StreetMind* also contributes to understanding health-related social dynamics. Features such as mobility tracking and social context tagging offer potential to detect deviations from behavioral baselines that may signal symptom onset or relapse, particularly in psychiatric populations. These data open new opportunities for early intervention strategies rooted in behavioral signatures. While internal (mind) and external (environment) structures typically change slowly in health, private, and public spaces, they can also rapidly shift due to significant internal and external “emergency” events [58]. *StreetMind* supports detecting both short-term and long-term fluctuations in these health–environment interactions, helping characterize self-perceived “health spaces” and their relationship to specific environmental areas and features.

Moreover, *StreetMind* supports detailed mapping of person–place interaction over time. Through the integration of geospatial, affective, and behavioral data, it facilitates the identification of personal “health spaces” and their alignment with built or natural environments. Such personalized environmental fingerprints resemble emerging concepts in network neuroscience [59] and link well with studies that associate architectural form and mobility with brain function [59, 60]. The data from the *StreetMind* platform will enable deeper analysis by incorporating the observed personal networks and interactions alongside the logged daily experiences of users, thereby providing maps of mental health. Theoretically, it considers cognitive processes like attention and memory, which serve as primary mechanisms in learning processes, all key for mental health and integration of environmental exposures. It further contributes to the understanding of the triggers underlying symptom-related behaviors and relapse. For example, individuals with alcohol use disorder often choose abstinence due to the harmful consequences of excessive drinking. However, encountering situations previously linked to alcohol use can prompt a relapse, highlighting individual and multidimensional contexts and their sensitivity to personal factors [61–64].

Expanding beyond psychological data, *StreetMind* is designed for integrative analysis alongside physiological, molecular, cognitive, and behavioral datasets. This multimodal approach can deepen our understanding of the interplay between external exposures and internal psychological and biological states, contributing to the development of predictive models in mental health research. *StreetMind* also complements wearable technologies by identifying location-specific patterns of activity and supporting context-aware feedback on physical health and well-being [65]. Ultimately, *StreetMind*’s adaptability ensures that it can be scaled and refined to meet the needs of various populations, advancing personalized and community-centered health strategies. This also relates to the promotion of a tailored community engagement and stakeholder [66] fostering trust, facilitating exchanges and change in behavior, and reflecting the viewpoints of those most impacted by health decisions.

The modular architecture of *StreetMind* allows adaptation across a range of cohorts, use cases, and study designs – from prevention and early intervention to public health monitoring. Its real-time, context-sensitive data streams can inform timely behavioral feedback and early risk detection strategies, supporting scalable, personalized public health interventions. The inclusion of publicly visible spots, real-time updates, and interactive user features also fosters dialogue between citizens and researchers, enabling data-driven insights that reflect lived experience.

In the context of climate change and health, *StreetMind* provides a unique opportunity to generate bottom-up indicators of environmental resilience. It allows for the evaluation of emotional and behavioral adaptation in response to changing environmental conditions, potentially informing policy on climate-sensitive health systems [67–69]. For example, longitudinal shifts in safety perception between urban and rural environments during periods of societal instability could be tracked and related to changing needs for community infrastructure or services.

Local associations, regional actors, and decision-makers need information about the realities of living in their region. *StreetMind* is designed for long-term use by the community and multiple cohorts. As spots can be updated and new users can rate existing spots and add their own, we will also be able to track the temporal development of an environmental area, such as a city, and the behaviors of individuals along these developments. *StreetMind* allows for assessing the opinion and perception of citizens of their region, together with their health and related factors, and can thereby facilitate a dialogue between citizens, scientists, and decision-makers to enhance informed policies and to evaluate them on an ongoing basis.

Some, but not all, outlook potentials relate to feedback-informed community prevention. *StreetMind* has the potential to serve as a tool for psychological prevention strategies to assess, model, and predict severity and treatment, including real-time data of a client’s maladaptive behaviors and mobility patterns, mood, contextual information (geo-positioning and environmental exposures), or social interactions [70].

*StreetMind*’s capacity to support both individual- and community-level profiling makes it highly relevant for urban planning, health equity initiatives, and real-time environmental evaluation. As areas evolve and user input accumulates, it becomes possible to monitor the dynamic development of urban spaces and their social–emotional resonance over time. It further informs feedback loops, including predictive and normative modeling, between individuals – for example, also between clinicians and patients in precision psychotherapy [66], to optimize behavioral efficacy.

### Statistical approaches and study designs for StreetMind data

The *StreetMind* platform produces complex, high-dimensional, and temporally structured data. To leverage its full analytical potential, tailored statistical and computational strategies are required. These include the following: Multilevel and mixed-effects models to account for repeated measures nested within individuals and to explore within- and between-person variability; time-series analyses for capturing dynamic changes in emotion, behavior, or environmental context across hours or days; network analyses to model co-occurrence of emotions, environmental perceptions, and health outcomes across spatial and temporal dimensions; spatial statistics (e.g., spatial autocorrelation and kernel density estimation) to detect and interpret geographic patterns in subjective and objective experiences; and machine learning approaches to identify nonlinear patterns or predictors of mental health from integrated sensor, survey, and map-based data.

In terms of study designs, *StreetMind* supports the following: Cross-sectional snapshots for mapping real-time experiences in urban versus rural environments; longitudinal cohort studies for detecting changes in environmental exposures and their impact over time; *N*-of-1 and idiographic studies, allowing personalized assessments and micro-intervention trials; and community-based participatory research designs, particularly valuable for citizen science and stakeholder engagement. These approaches enable *StreetMind* to contribute to both population-level inference and individual-level precision health strategies.

### Limitations and ethical considerations

While *StreetMind* provides valuable tools for integrating subjective and objective experiences across geography, technology, and mental health, certain limitations must be considered. One key issue is the potential for digital exclusion. Individuals from older age groups, those with physical or cognitive impairments, or those with limited technological literacy may face barriers in using the app effectively. Similarly, access to smartphones or consistent internet connectivity is not universal and may limit representativeness in lower socioeconomic or rural populations.

Cultural variability also poses a challenge, particularly with regard to data sharing norms and privacy expectations. What is perceived as acceptable app-based self-disclosure in one sociocultural context may be viewed as intrusive in another. Thus, app adoption and engagement may vary significantly across populations and regions.

To address these concerns, *StreetMind* is designed to support inclusive adaptation strategies. These include adjustable app features (e.g., simplified interfaces, font scaling and audio-assisted inputs), platform availability in multiple languages, and granular privacy controls to ensure users maintain agency over their shared data. Additionally, ongoing user co-design sessions and accessibility audits are planned to guide further development. These steps aim to ensure that *StreetMind* serves as a socially responsive and ethically grounded tool for diverse users.

## Conclusions and contributions

In summary, advancements in digital technologies allow for detailed assessments with increased temporal, spatial, and individual granularity to capture daily life behaviors. *StreetMind* leverages the progress in digital technologies and citizen science for detailed assessments with increased temporal, spatial, and individual granularity to capture daily life behaviors at the intersection of health and environment. By combining behavioral traces with psychological characteristics and both active and passive environmental information, it provides insights into if, why, and how individual’s behaviors, feelings, and thoughts are influenced by various – situational and social – factors. By combining high-resolution geodata with survey data, it enables research on environmental stressors [71, 72] to inform data-driven health policies and interventions. It can also be used to advance urban planning and transport design models to predict activity patterns linked to mental health outcomes. *StreetMind’s flexible design allows for scalable use* in various research settings and studies (for first cohorts, see, e.g., https://www.streetmind.eu/blog), fostering a network of user-contributed insights on their communities and surroundings, emotions, thoughts, and behaviors. This community model can foster a real-time exchange of information and engagement, and well-being hotspots offering valuable insights for researchers and stakeholders, and the public.

## Supporting information

10.1192/j.eurpsy.2025.10107.sm001 Nees et al. supplementary material Nees et al. supplementary material

## Data Availability

Data are made visible in the app and website – for example, through the integrated map structure, if participants have agreed to share their entries. Raw data will only be shared with investigators whose proposed use of the data is provided in a methodologically sound proposal and data will only be shared to achieve aims in the approved proposal.

## Supplementary material

The supplementary material for this article can be found at http://doi.org/10.1192/j.eurpsy.2025.10107.

## Use of *StreetMind* in projects and initiatives

Proposals should be directed to the corresponding author F.N. To establish a respective pipeline within *StreetMind*, a data sharing and transfer agreement, as well as ethic approval, are needed.
