# Supplementary material for: Comprehensive perceptions at the interface between health and environment: Applications models with a citizen science tool
Source: Eur Psychiatry. 2025 Nov 20;68(1):e165. doi: 10.1192/j.eurpsy.2025.10107 (PMC12930206; doi:10.1192/j.eurpsy.2025.10107)

**Supplementary Material**

**1.1 Privacy, data protection and data management**

After account registration, participants are instructed to select a user role (e.g., a cohort name) from a dropdown-menu and enter in case of a cohort follow-up a unique identification number in the *StreetMind* app and at the beginning of the *StreetMind* survey to allow to link their stored and pseudonymised data to a specific cohort, or, in case of a not pre-defined community user, a fictional username. To use the app and fill in the web survey, participants create a platform-wide user account, with the minimum requirement of providing an email address, password, and name (real name or pseudonym). By doing this, participants agree with SPOTTERON’s terms of use (<https://www.spotteron.net/terms-of-use>) and privacy policy regulations (<https://www.spotteron.net/privacy>), which are in accordance with General Data Protection Regulations (GDPR) of the European Union (EU). We consider the necessary ethical and legal rules for digital tools, including protecting privacy, preserving autonomy, avoiding discrimination (Gasser et al., 2020). Personal data is stored separately from research data gathered in the app and survey.

**1.2 Citizen Science features**

There are various functionalities for participants to interact with the created data, by e.g., choosing between different map types, and receiving individual feedback via graphs. Heat maps, for example, visualizes data through a two-dimensional domain (such as points on a wall or a map) projected onto a numerical scale (like a thermometer’s scale) and displayed with color that represent different levels and values along a respective scale such as valence ratings. This visualization method is intended to offer a quick, intuitive overview of large datasets, making it easy to identify particularly significant values at a glance. This can be mapped based on all information provided in the app, and can thus show sensitive areas in terms of health and well-being, social or physical activity, environmentally negative characteristics such as increased perceived exposure to noise or positive characteristics such as perceived exposure to nature or relaxing atmosphere, and whether and how this changes over time. On the individual level, heat maps for example highlight a person`s most pleasant and preferred places and activities, again also how this changes over time, and those with the highest well-being, which does not necessarily need to correlate. Specifically, if these correlations are not strong, this provides important information for both the individual and the community as well as the researcher and clinicians as to why efforts to maintain or increase health and prevent illness might fail and how these might need to be adapted. The community can so also learn more about their own and other places and areas, and information can be used by expert stakeholders from different health and environmental sectors.

**Suppl. Tables**

**Suppl. Table 2.** StreetMind survey: overview of psychometric instruments

| Sections | Instruments | Estimated duration | Source |
| --- | --- | --- | --- |
| General Information | DE/SES | 2 MIN | adapted from Lambert et al., 2018 |
| Urbanicity, Living conditions | Urbanicity + Country of Residency + ZIP code | 2 MIN | Lederbogen et al., 2011 |
|  | Eco-emotions & behaviour | 4 MIN | Stanley et al., 2021 |
| Influence of  current affairs | Ukraine war items | 1 MIN | adapted from COSMO report* |
|  | CRISIS (subscales) | 4 MIN | Nikoladidis et al., 2021 |
| Social aspects | F_SozU_K14 + WHO2.0 (single item) | 3 MIN | Fydrich et al., 2009; WHO, 1988 |
|  | FAD (subscale) | 2 MIN | Boterhoven et al., 2015 |
|  | SSS | 1 MIN | Hoebel et al., 2015 |
| Personality | NEO-FFI | 7 MIN | Costa & McCrae, 1992 |
|  | TCI_R (subscales) | 5 MIN | Pelissolo et al., 2005; Richter et al., 1999 |
| Physical health | CSI (single items; physical symptoms) | 1 MIN | Mayer et al., 2012 |
|  | SIMPAQ (physical activity) | 3 MIN | Rosenbaum et al., 2016, 2020 |
| Mental health | BSI (subscales: anxiety, phobia, anhedonia) | 3 MIN | Derogatis et al., 1988 |
|  | PHQ9 (depression) | 1 MIN | Beard et al., 2016 |
|  | MPFI24 (psychological flexibility) | 3 MIN | Rolffs et al., 2018 |
|  | MEQ (chronotype) | 5 MIN | Terman et al., 2001 |
|  | UCLA (loneliness) | 3 MIN | Russell, 1996 |
|  | STAI_T (trait anxiety) | 2 MIN | Spielberger, 1983 |
|  | AUDIT (Alcohol use) | 3 MIN | Saunders et al., 1993 |
|  | FTND (smoking) | 2 MIN | Etter, 2008 |
|  | ESPAD (single items; drug use) | 1 MIN | Kraus et al., 2016 |
|  | ASRS (ADHD) | 4 MIN | Kessler et al., 2005 |
|  | LEQ-82 (life experiences) | 15 MIN | Sarason et al., 1978; Norbeck et al., 1983 |
|  | CTS (childhood trauma) | 2 MIN | Lang et al., 2018 |
|  | PSS10 (perceived stress) | 2 MIN | Cohen et al., 1994 |
|  | EDEQ (eating disorder) | 5 MIN | Mond et al., 2004 |
| Motivation & Impulsivity | BIS/BAS (motivation) | 4 MIN | Jorm et al., 1998 |
|  | BIS15 (impulsivity) | 2 MIN | Spinella, 2007 |
|  | TOTAL | **~70-80 MIN** |  |

**Abbreviations:** ASRS – Adult ADHD Self-Report Scale; AUDIT – Alcohol Use Disorders Identification Test; BIS/BAS – Behavioural Inhibition System/ Behavioral Approach System; BIS15 – Barratt Impulsiveness Scale; BSI – Brief Symptom Inventory; CRISIS – CoRonavIruS Health Impact Survey; CSI – Central Sensitization Inventory; CTS – Childhood Trauma Screener; DE – Demographic Information; EDEQ – Eating Disorder Examination Questionnaire; ESPAD – European School Survey Project on Alcohol and Other Drugs; F_SozU_K14 – Fragebogen Sozialer Unterstützung (Questionnaire regarding social support); LEQ – Life Event Questionnaire; MEQ – Morning-Evening Questionnaire; MPFI24 – Multidimensional Psychological Flexibility Inventory; NEO-FFI – NEO Five Factor Inventory; PHQ9 – Prime MD Patient Health Questionnaire; PSS – Perceived Stress Scale; SES – Socio-economic status; SIMPAQ – Simple Physical Activity Questionnaire; SSS – Subjective Social Status; STAI_T – Trait anxiety Inventory; TCI-R – Temperament and Character Inventory; WHO – World Health Organization;

*COSMO – Covid-19 Snapshot Monitoring: https://projekte.uni-erfurt.de/cosmo2020/files/COSMO_W61.pdf

**Suppl. Table 3.** Overview on Packages and Libraries used for visualizations.

| **Language** | **Usage** | **Package/Library** | **Version** | **Citation** |
| --- | --- | --- | --- | --- |
| R (R Core Team, 2022) | Plotting | *ggplot2* | 3.4.2 | (Wickham, 2016) |
|  |  | *ggridges* | 0.5.4 | (Wilke, 2022) |
|  |  | *ggradar* | 0.2.0 | (Bion, 2023) |
|  |  | *ggpubr* | 0.6.0 | (Kassambara, 2023) |
|  |  | *circlize* | 0.4.15 | (Gu et al., 2014) |
|  |  | *hrbrthemes* | 0.8.0 | (Rudis, 2020) |
|  |  | *ggmap* | 3.0.2 | (Kahle & Wickham, 2013) |
|  | Spatial Data | *sf* | 1.9 | (Pebesma, 2018) |
|  |  | *geojsonsf* | 2.0.3 | (Cooley, 2022) |
|  |  | *geosphere* | 1.18 | (Hijmans, 2022) |
|  | Data Processing | *tidyverse* | 1.3.2 | (Wickham et al., 2019) |
|  |  | *dplyr* | 1.1.2 | (Wickham et al., 2023) |
|  |  | *reshape2* | 1.4.4 | (Wickham, 2007) |
|  |  | *lessR* | 4.2.9 | (Gerbing, 2021) |
|  |  | *rstudioapi* | 0.14 | (Ushey et al., 2022) |
|  | Data Analysis | *psych* | 1.9.12 | (Revelle, 2019) |
|  |  | *zoo* | 1.11 | (Zeileis & Grothendieck, 2005) |
|  |  | *RcppRoll* | 0.3.0 | (Ushey, 2018) |
| Python 3.11 | Network Analysis | *NetworkX* | 3.1 | (Hagberg et al., 2008) |
|  |  | *OSMnx* | 1.6.0 | (Boeing, 2017) |

**References**

Beard, C., Hsu, K. J., Rifkin, L. S., Busch, A. B., & Björgvinsson, T. (2016). Validation of the PHQ-9 in a psychiatric sample. *Journal of affective disorders*, *193*, 267-273.

Bion, R. (2023). *ggradar: Create radar charts using ggplot2*.

Boeing, G. (2017). OSMnx: New methods for acquiring, constructing, analyzing, and visualizing complex street networks. *Computers, Environment and Urban Systems*, *65*, 126–139.

Boterhoven de Haan, K. L., Hafekost, J., Lawrence, D., Sawyer, M. G., & Zubrick, S. R. (2015). Reliability and validity of a short version of the general functioning subscale of the McMaster Family Assessment Device. *Family process*, *54*(1), 116-123.

Cohen, S., Kamarck, T., & Mermelstein, R. (1994). Perceived stress scale. *Measuring stress: A guide for health and social scientists*, *10*(2), 1-2.

Cooley, D. (2022). *geojsonsf: GeoJSON to Simple Feature Converter*. https://CRAN.R-project.org/package=geojsonsf

Costa Jr, P. T., & McCrae, R. R. (1992). Four ways five factors are basic. *Personality and individual differences*, *13*(6), 653-665.

Derogatis, L. R., & Melisaratos, N. (1983). The brief symptom inventory: an introductory report. *Psychological medicine*, *13*(3), 595-605.

Etter, J. F. (2008). Comparing the validity of the cigarette dependence scale and the Fagerström test for nicotine dependence. *Drug and alcohol dependence*, *95*(1-2), 152-159.

Fydrich, T., Sommer, G., Tydecks, S., & Brähler, E. (2009). Fragebogen zur sozialen unterstützung (F-SozU): Normierung der Kurzform (K-14). Zeitschrift für medizinische Psychologie, 18(1), 43-48.

Gerbing, D. W. (2021). Enhancement of the Command-Line Environment for use in the Introductory Statistics Course and Beyond. *Journal of Statistics and Data Science Education*, *29*(3), 251–256. https://doi.org/10.1080/26939169.2021.1999871

Gu, Z., Gu, L., Eils, R., Schlesner, M., & Brors, B. (2014). circlize implements and enhances circular visualization in R. *Bioinformatics*, *30*(19), 2811–2812.

Hagberg, A., Swart, P., & S Chult, D. (2008). *Exploring network structure, dynamics, and function using NetworkX*. Los Alamos National Lab.(LANL), Los Alamos, NM (United States).

Hijmans, R. J. (2022). *geosphere: Spherical Trigonometry*. https://cran.r-project.org/package=geosphere

Hoebel, J., Müters, S., Kuntz, B., Lange, C., & Lampert, T. (2015). Messung des subjektiven sozialen Status in der Gesundheitsforschung mit einer deutschen Version der MacArthur Scale

Jorm, A. F., Christensen, H., Henderson, A. S., Jacomb, P. A., Korten, A. E., & Rodgers, B. (1998). Using the BIS/BAS scales to measure behavioural inhibition and behavioural activation: Factor structure, validity and norms in a large community sample. *Personality and individual Differences*, *26*(1), 49-58.

Kahle, D., & Wickham, H. (2013). ggmap: Spatial Visualization with ggplot2. *The R Journal*, *5*(1), 144–161. https://journal.r-project.org/archive/2013-1/kahle-wickham.pdf

Kassambara, A. (2023). *ggpubr: “ggplot2” Based Publication Ready Plots*. https://cran.r-project.org/package=ggpubr

Kessler, R. C., Adler, L., Ames, M., Demler, O., Faraone, S., Hiripi, E. V. A., ... & Walters, E. E. (2005). The World Health Organization Adult ADHD Self-Report Scale (ASRS): a short screening scale for use in the general population. *Psychological medicine*, *35*(2), 245-256.

Kraus, L., & Nociar, A. (2016). *ESPAD report 2015: results from the European school survey project on alcohol and other drugs*. European Monitoring Centre for Drugs and Drug Addiction.

Lampert, T., Hoebel, J., Kuntz, B., Müters, S., & Kroll, L. E. (2018). Socioeconomic status and subjective social status measurement in KiGGS Wave 2. *Journal of Health Monitoring*, *3*(1), 108.

Lang, J. M., & Connell, C. M. (2018). The child trauma screen: A follow‐up validation. *Journal of traumatic stress*, *31*(4), 540-548.

Lederbogen, F., Kirsch, P., Haddad, L., Streit, F., Tost, H., Schuch, P., ... & Meyer-Lindenberg, A. (2011). City living and urban upbringing affect neural social stress processing in humans. *Nature*, *474*(7352), 498-501.

Mayer, T. G., Neblett, R., Cohen, H., Howard, K. J., Choi, Y. H., Williams, M. J., ... & Gatchel, R. J. (2012). The development and psychometric validation of the central sensitization inventory. *Pain Practice*, *12*(4), 276-285.

Mond, J. M., Hay, P. J., Rodgers, B., Owen, C., & Beumont, P. J. (2004). Validity of the Eating Disorder Examination Questionnaire (EDE-Q) in screening for eating disorders in community samples. *Behaviour research and therapy*, *42*(5), 551-567.

Nikolaidis, A., Paksarian, D., Alexander, L., Derosa, J., Dunn, J., Nielson, D. M., Droney, I., Kang, M., Douka, I., Bromet, E., Milham, M., Stringaris, A., & Merikangas, K. R. (2021). The Coronavirus Health and Impact Survey (CRISIS) reveals reproducible correlates of pandemic-related mood states across the Atlantic. Scientific Reports, 11(1). <https://doi.org/10.1038/s41598-021-87270-3>

Norbeck, J. S., Lindsey, A. M., & Carrieri, V. L. (1983). Further development of the Norbeck Social Support Questionnaire: normative data and validity testing. *Nursing research*, *32*(1), 4-9.

Pebesma, E. (2018). Simple Features for R: Standardized Support for Spatial Vector Data. *The R Journal*, *10*(1), 439–446. https://doi.org/10.32614/RJ-2018-009

Pelissolo, A., Mallet, L., Baleyte, J. M., Michel, G., Cloninger, C. R., Allilaire, J. F., & Jouvent, R. (2005). The Temperament and Character Inventory‐Revised (TCI‐R): psychometric characteristics of the French version. *Acta Psychiatrica Scandinavica*, *112*(2), 126-133.

Revelle, W. (2019). *psych: Procedures for Psychological, Psychometric, and Personality Research*. https://cran.r-project.org/package=psych

Richter, J., Eiseman, M., Richter, G., & Cloninger, C. (1999). Das Temperament und Charakter Inventar: Ein Leitfaden über seine Entwicklung und Anwendung [German edition of the original TCI manual].

Rolffs, J. L., Rogge, R. D., & Wilson, K. G. (2018). Disentangling components of flexibility via the hexaflex model: Development and validation of the Multidimensional Psychological Flexibility Inventory (MPFI). *Assessment*, *25*(4), 458-482.

Rosenbaum, S., & Ward, P. B. (2016). The simple physical activity questionnaire. *The Lancet Psychiatry*, *3*(1), e1.

Rosenbaum, S., Morell, R., Abdel-Baki, A., Ahmadpanah, M., Anilkumar, T. V., Baie, L., ... & Ward, P. B. (2020). Assessing physical activity in people with mental illness: 23-country reliability and validity of the simple physical activity questionnaire (SIMPAQ). *BMC psychiatry*, *20*(1), 1-12.

Rudis, B. (2020). hrbrthemes: Additional themes, theme components and utilities for “ggplot2.” *Hrbrthemes Documentation. Available Online: Https://Rdrr. Io/Cran/Hrbrthemes (Accessed on 26 March 2020)*.

Russell, D. W. (1996). UCLA Loneliness Scale (Version 3): Reliability, validity, and factor structure. *Journal of personality assessment*, *66*(1), 20-40.

Sarason, I. G., Johnson, J. H., & Siegel, J. M. (1978). Assessing the impact of life changes: development of the Life Experiences Survey. *Journal of consulting and clinical psychology*, *46*(5), 932.

Saunders, J. B., Aasland, O. G., Babor, T. F., De la Fuente, J. R., & Grant, M. (1993). Development of the alcohol use disorders identification test (AUDIT): WHO collaborative project on early detection of persons with harmful alcohol consumption‐II. *Addiction*, *88*(6), 791-804.

Spielberger, C. D. (1983). State-trait anxiety inventory for adults.

Spinella, M. (2007). Normative data and a short form of the Barratt Impulsiveness Scale. *International Journal of Neuroscience*, *117*(3), 359-368.

Stanley, S. K., Hogg, T. L., Leviston, Z., & Walker, I. (2021). From anger to action: Differential impacts of eco-anxiety, eco-depression, and eco-anger on climate action and wellbeing. The Journal of Climate Change and Health, 1, 100003.

Terman, M., Rifkin, J. B., Jacobs, J., & White, T. M. (2001). Morningness-eveningness questionnaire (Revised). *New York, NY: New York State Psychiatric Institute*.

Ushey, K. (2018). *RcppRoll: Efficient Rolling / Windowed Operations*. https://cran.r-project.org/package=RcppRoll

Ushey, K., Allaire, J. J., Wickham, H., & Ritchie, G. (2022). *rstudioapi: Safely Access the RStudio API*. https://cran.r-project.org/package=rstudioapi

Wickham, H. (2007). Reshaping Data with the {reshape} Package. *Journal of Statistical Software*, *21*(12), 1–20. http://www.jstatsoft.org/v21/i12/

Wickham, H. (2016). *ggplot2: Elegant Graphics for Data Analysis*. Springer-Verlag New York. https://ggplot2.tidyverse.org

Wickham, H., Averick, M., Bryan, J., Chang, W., McGowan, L., François, R., Grolemund, G., Hayes, A., Henry, L., Hester, J., Kuhn, M., Pedersen, T., Miller, E., Bache, S., Müller, K., Ooms, J., Robinson, D., Seidel, D., Spinu, V., … Yutani, H. (2019). Welcome to the Tidyverse. *Journal of Open Source Software*, *4*(43), 1686. https://doi.org/10.21105/joss.01686

Wickham, H., François, R., Henry, L., Müller, K., & Vaughan, D. (2023). *dplyr: A Grammar of Data Manipulation*. https://cran.r-project.org/package=dplyr

Wilke, C. O. (2022). *ggridges: Ridgeline Plots in “ggplot2.”* https://cran.r-project.org/package=ggridges

World Health Organization. (‎1988)‎. WHO psychiatric disability assessment schedule (‎WHO/DAS: with a guide to its use. World Health Organization. https://apps.who.int/iris/handle/10665/40429

Zeileis, A., & Grothendieck, G. (2005). zoo: S3 Infrastructure for Regular and Irregular Time Series. *Journal of Statistical Software*, *14*(6), 1–27. https://doi.org/10.18637/jss.v014.i06

**Supplementary Table 1 (excel file)**


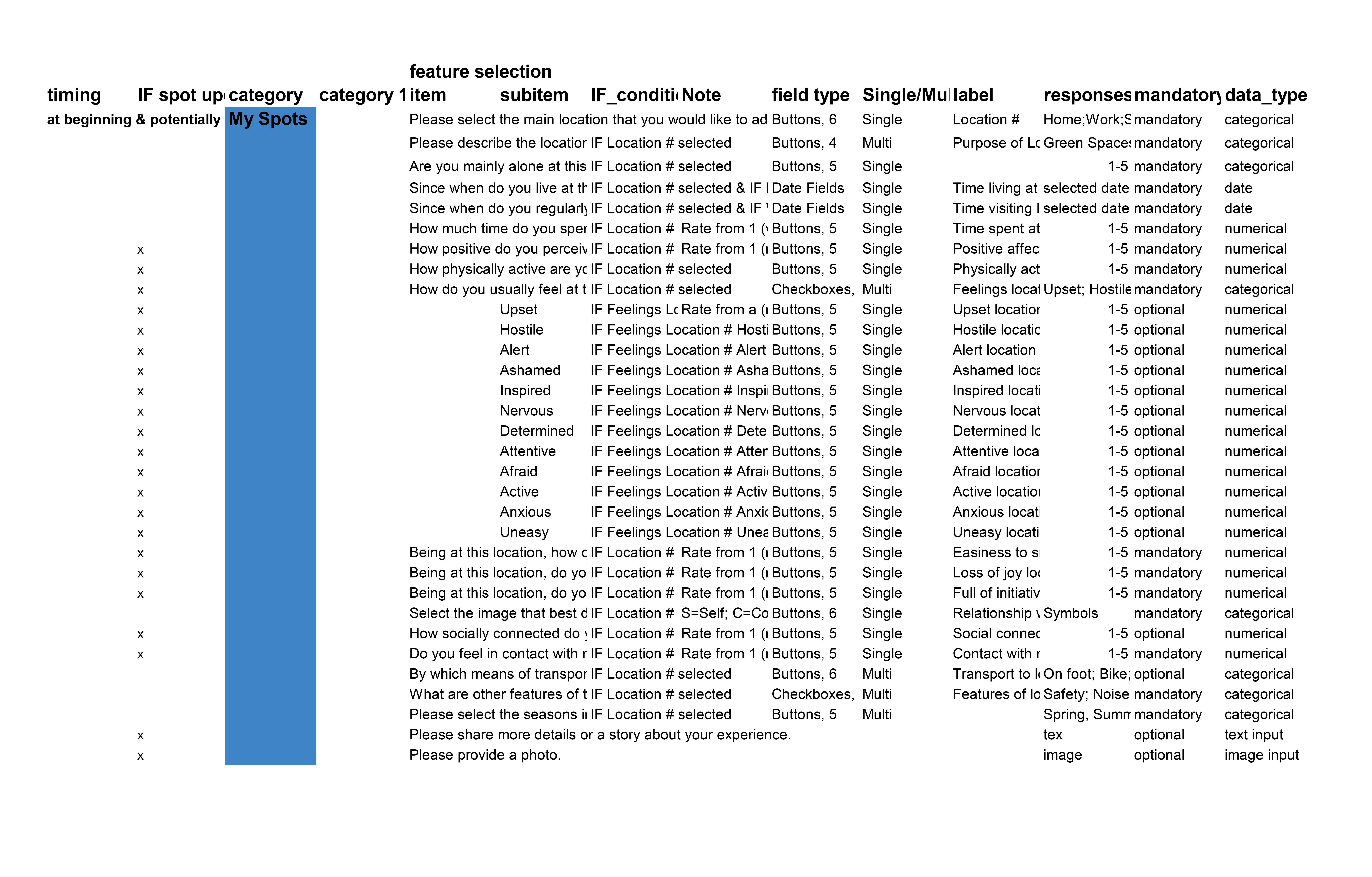


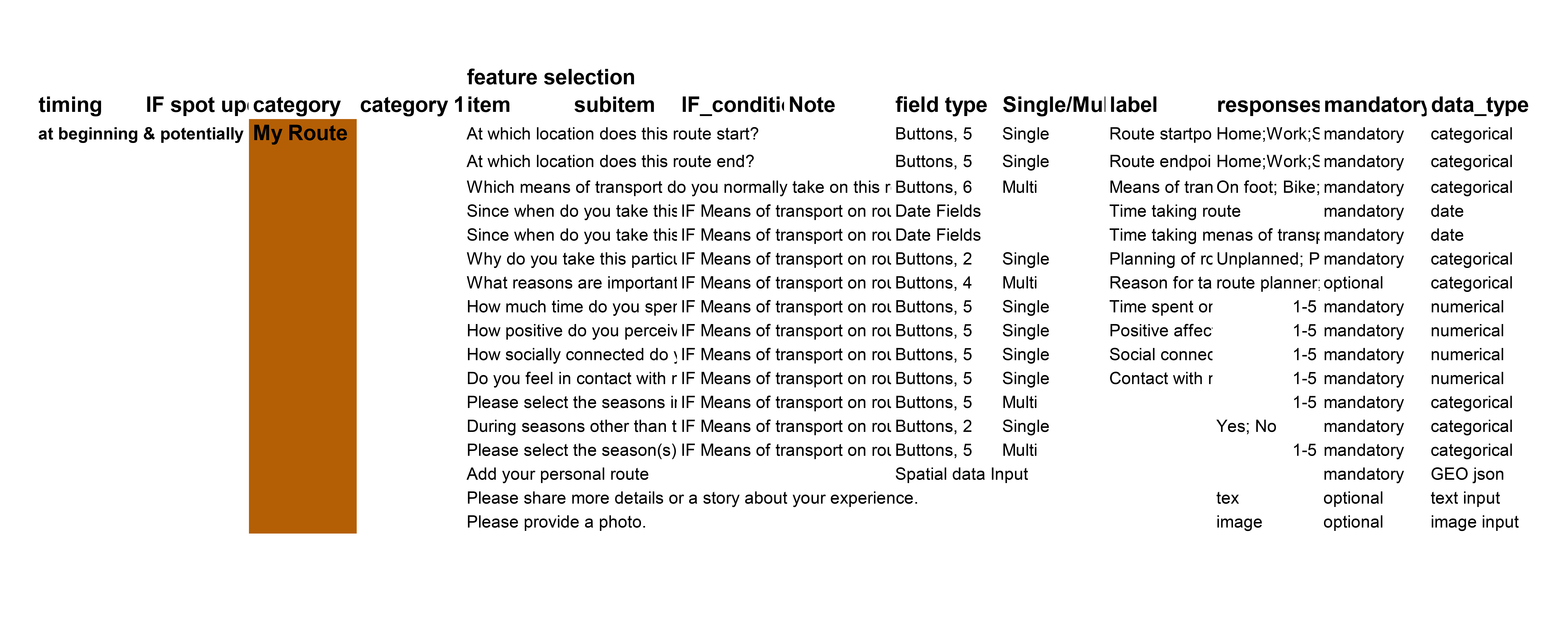

Supplement: Nees et al. supplementary material [file S0924933825101077sup001.docx]
